# Development assistance for health given to Nepal by China and India: a comparative study

**DOI:** 10.1186/s12992-014-0076-6

**Published:** 2014-11-19

**Authors:** Haomin Yang, Shambhu Prasad Acharya, Peilong Liu, Yan Guo

**Affiliations:** Department of Health Policy and Management, School of Public Health, Peking University, No. 38 Xueyuan Road, Haidian District, Beijing, 100191 China; Department of Country Cooperation and Collaboration with the UN System, World Health Organization, Avenue Appia 20, 1211 Geneva 27, Switzerland; Department of Global Health, School of Public Health, Peking University, No. 38 Xueyuan Road, Haidian District, Beijing, 100191 China

**Keywords:** China, India, Nepal, Development Assistance for Health

## Abstract

**Background:**

Development assistance for health (DAH) promotes health development in low and middle income countries. China and India, as emerging donors, have scaled-up their DAH programs during the recent years. Nepal, as a neighboring country to China and India, has witnessed the history and development of China’s and India’s DAH.

**Methods:**

This research uses a literature review and in-depth individual interviewing to compare the history and forms of DAH given from China and India to Nepal.

**Results:**

During 60-years of DAH to Nepal, China and India have gradually increased the scale and forms of DAH, and both focus on dispatching medical teams or faculty, building health facilities and gifting medicines and equipment. However, the inclusiveness of Nepalese culture, diplomatic interests, and Nepal’s cultural, linguistic and geographical closeness to India make the DAH of India different from that of China. India’s DAH also includes support to grass roots NGOs and public health interventions.

**Conclusion:**

China’s and India’s insistence on a recipient-driven mechanism keeps the aid programs aligned with Nepal’s health development plan and respects Nepal’s “ownership”. China can learn from India to start the development assistance for health related NGOs and public health intervention.

## Background

Development assistance for health (DAH) is an important source of health expenditure in low and middle income countries [1]. DAH has increased from 6.47 billion USD in 2000 to 19.9 billion USD in 2012, accounting for 6.7% and 11.14% of the total amount of official development assistance (ODA) given [2]. DAH is not only a tool to protect national non-traditional security, especially public health security [3], but also an approach to promoting global health equity. Low income countries need external development assistance as their level of health financing cannot meet the challenges they will face [4]. Developed countries and the international community can also achieve the goal of equity in health financing and resource allocation through DAH [5].

With the persistent financial crisis, especially in OECD countries, in recent years, traditional donors have partly reduced their donations, but some emerging donors have started to shoulder more international obligations and to invest more in global health, especially China and India [6]. Both China and India started DAH in the 1950s when they just established the new republics and were very poor themselves. Becoming the emerging economics in the world, China and India have expanded the scale of DAH in recent years.

China’s DAH focuses on the low income developing countries, especially those from Africa and Asia. Currently, there are 1171 doctors providing health care services in 49 developing countries [7]. In Africa, China has completed 33 hospitals and 30 anti-malaria centers, and donated 80 million USD for medicines and equipment in these hospitals and centers since 2006 [8]. In Asia, China is continuously improving the regional health cooperation mechanisms with the investment of 82 thousand USD in the Greater Mekong sub-region and Central Asia [9]. China has also donated 9 hospitals for neighboring countries such as Nepal, Afghanistan and Pakistan.

India’s DAH mainly goes to the south Asian neighboring countries and Africa. India has helped the South Asian countries to construct 8 large hospitals and many clinics and provided remote medical services for 42 African countries. In addition, India also dispatches doctors to Afghanistan and Maldives, and has implemented public health interventions in Nepal and Bhutan [6].

The growth of China’s and India’s DAH has attracted global attention. Ann Florini et al. found China and India’s approaches of DAH different from those adopted by developed nations. They framed their DAH programs overwhelmingly in bilateral terms, focused around infrastructure and technical assistance, and not well connected to global health initiatives [10]. Yanzhong Huang compared the motivation and coordination mechanisms of China’s and India’s DAH, indicating their DAH serves national interest and humanitarian needs, although lacks institutional capacities [11]. However, neither of these papers compared the differences between China and India in a selected recipient.

This research aims to compare the DAH given by China and India to Nepal to fill in this blank. It will compare the histories and forms of DAH given by China and India to Nepal to discover the similarities and differences between these two typical non-traditional donors in terms of their DAH to Nepal. With a comprehensive understanding of China’s and India’s contribution to Nepal’s health sector, we will explore the relative different political, economic or cultural causes determining both countries’ activities in Nepal, as well as the influences from Nepal that come in return. This article will also conclude with some lessons from China and India’s experience of DAH to Nepal and thus contribute more to global health contours.

## Method

This research will use a literature review and in-depth individual interviewing to gain the materials required for comparison. The literature comes from websites and reports by China’s embassy in Nepal, India’s embassy in Nepal, the Ministry of Finance, the Ministry of Health and Population of Nepal and other official websites of Chinese and Indian government and official news agencies, as well as annual reports on the programs. We used the advanced search engine of Google and Baidu to search for the information in the those websites with key words of “China”, “India”, “Nepal”, “health”, “aid” and “assistance”, both in Chinese and English. Reports of the programs and several paper archives are scanned by the author to discover useful information. Although the data quality is mixed, these data covered all China’s and India’s DAH programs in Nepal and confirmed by official. Actually, the Chinese, Indian and Nepalese governments and the project organizations are essentially the source of all the data, without other independently confirmed sources. Nevertheless, the information generated here is believed to be the most robust and double check for consistency between the donor party and the recipient party. Some of the funding estimates may not be an exact number but actually reflects the funding level of the project.

This research uses a snowball sampling method^a^ to find the officers in charge of China’s and India’s DAH programs. The primary key informants are China Medical Team leader in Nepal and director of Division of Policy Planning and International Cooperation in Nepalese Ministry of Health and Population. Twenty-one officers and health professionals in charge of and implementing China’s and India’s DAH programs in Nepal and 4 managers of other development partners in Nepal accepted a semi-structural in-depth interview of 20 to 40 minutes. The interviews were taken from May to August, 2013. The interview structure includes history and current condition of each program, funding and trend, outcomes and effectiveness of DAH. Although there are limitations to using in-depth individual interviewing to obtain adequate unbiased and objective information, sufficiently detailed information can only be obtained by interviewing and carrying out spot observations as a result of the limited transparency of China’s and India’s programs.

## Results

### History of China’s and India’s DAH to Nepal

China began providing DAH to Nepal soon after building a diplomatic relationship with Nepal. In the 1950s and 1960s, China’s aid focused on medicine, vaccines and equipment for treating tuberculosis [12-14]. China’s annual donation stopped in 1973, however, for reasons unknown. From then on, China’s priority for development assistance to Nepal was economic development, “*mainly in terms of industry, roads and hydropower*” [government officer 20130729a1].

From 1996 on, China reinvested in the programs to provide DAH to Nepal with the construction of the B.P. Koirala Memorial Cancer Hospital and the dispatching of a medical team in 1999 [15]. Later, China built the Civil Service Hospital [16] and the National Ayurveda Research and Training Center [17] in Kathmandu, in 2006 and 2009, respectively and provides scholarships for Nepalese students to study medicine in China. China’s DAH programs focus on the functions of health systems, mainly facilities and capacity building, as well as service provision.

India’s first DAH program in Nepal was designed to support the development of Paropakar Prasuti Graha (Maternity Hospital) in 1959, including expansion of the building and the setting up of a pathological laboratory [18]. India also provided scholarships for Nepalese students to study in MBBS programs in India under the Colombo Plan in the 1950s and later with the Golden Jubilee Scholarship [19]. In 1973, India and Nepal agreed on an additional assistance program for the 1967/68-1974/75 assistance plan, during which time India constructed 12 clinics and started the Goiter Control Program, lasting for 25 years [20]. India strengthened the health system of Nepal with the expansion of Bir (national specialized) Hospital in 1985 and the construction of, as well as faculty support for, the B.P. Koirala Institute of Health Science (BPKIHS) from 1991. Shortly afterwards, India began donating several ambulances to Nepal annually, on the anniversaries of India’s Republic and Independence Days [21].

From 2000 on, India slightly reformed the DAH programs to support non-governmental organizations (NGOs) and local government, alongside the construction of tertiary hospitals, such as in the assistance given to the Nepal Netra Jyoti Sangh (Nepal Eye Foundation). The small development projects undertaken from 2003 onwards have helped India’s DAH connect with the essential health needs of the Nepalese through the construction of rural clinics and maternity centers. During this time, India has also finished the construction of a trauma center in Bir Hospital and a teaching building in BPKIHS [21].

**Figure 1:**
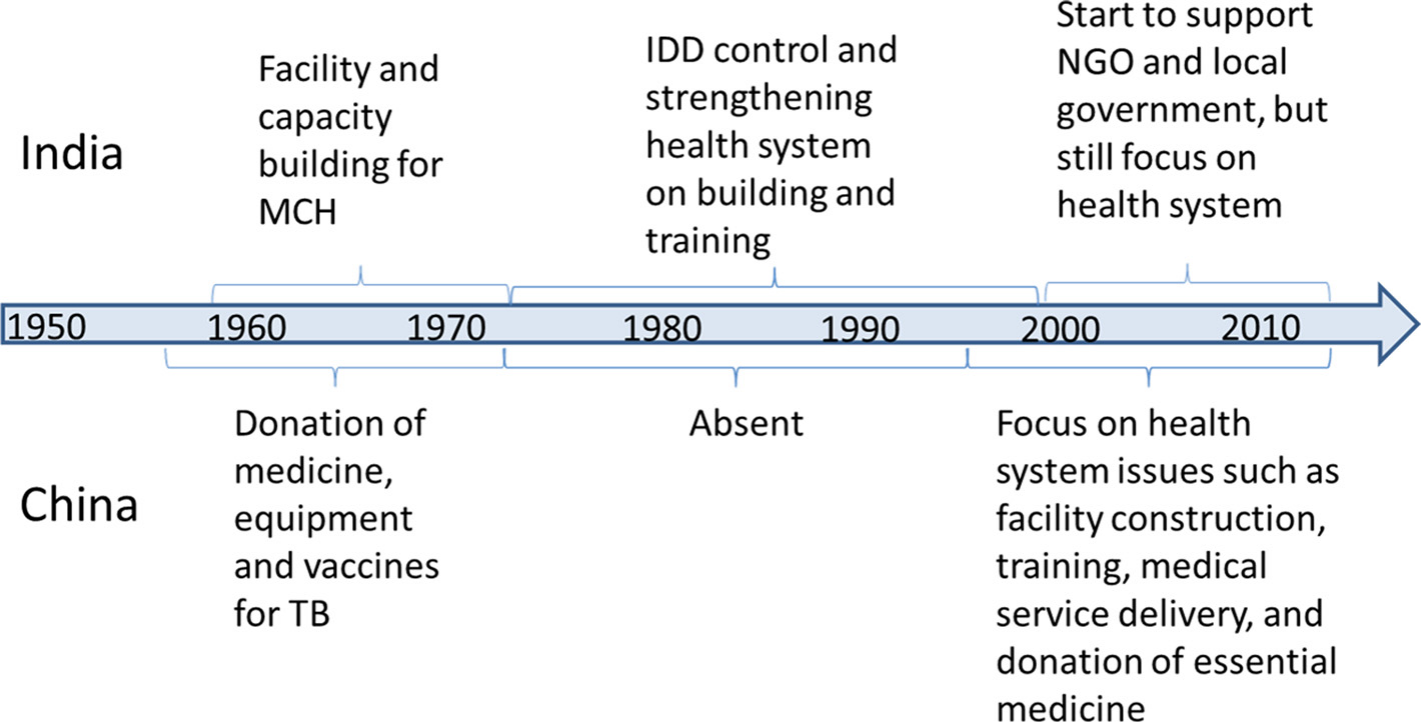
**Historic trend of China’s and India’s DAH in Nepal.**

**Figure 2:**
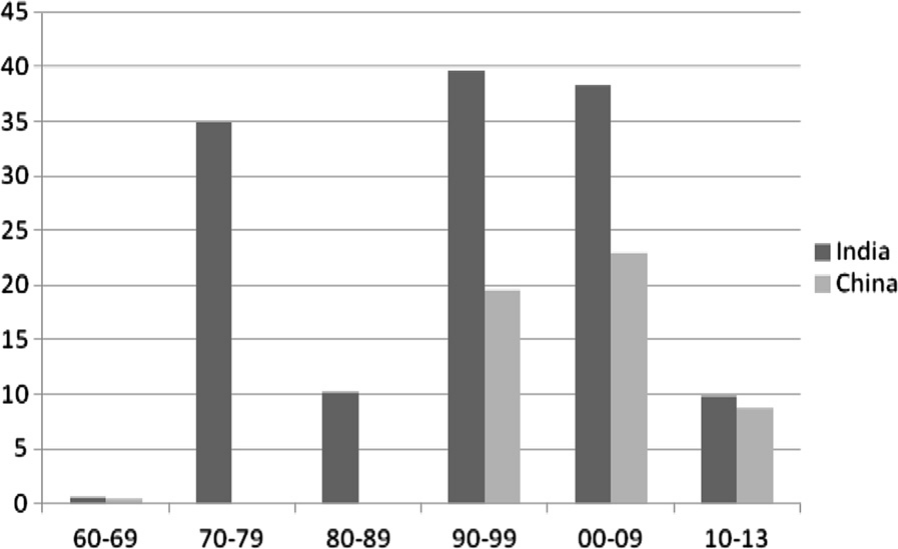
**Estimated monetary trends of DAH from China and India to Nepal (1960–2013) (in millions of USD, constant in 2010).** Source: Estimated and summarized from the funding of projects in Tables 1 and 2.

Figures 1 and 2 indicate the historic and monetary trends of China’s and India’s DAH to Nepal.

### Forms of China’s and India’s DAH programs in Nepal

China’s DAH to Nepal mainly focuses on facilities and capacity building, including dispatching medical teams, health facilities building work, training courses, donations of medicines and equipment, and scholarships. Table 1 lists different forms and the correspondent funding, period and outcome of China’s DAH programs. Considering the funding and efforts, it seems that a large amount of China’s DAH goes to dispatch of medical team and health facility building.

**Table 1:** **Forms of China’s DAH programs in Nepal**

**Forms**	**Name of project**	**Period**	**Funding**	**Output**
Dispatch of medical teams	China Medical Team in Cancer Hospital	1999~	Around 800,000 USD every year	143 members in 8 teams have worked in Nepal
Health facility building	B.P. Koirala Cancer Hospital	1996–1998	11.7 million USD	150-bedded hospital with the building area of 9850 m^2^
	Civil Service Hospital	2007–2009	Around 16 million USD	120-bedded hospital with the building area of 11330 m^2^ and 3 buildings
	National Ayurveda Research and Training Center	2009–2011	7.14 million USD	3 buildings with building area of 6436 m^2^
Training courses	Ayurveda Research and Training course	2013	Around 161,000 USD	19 officers and technicians trained
Donation of medicine and equipment	Medicine, vaccine and equipment related to TB	1956–1972	About 110,000 USD	8 batches of vaccines, medicines, equipment and money
	Medicine and equipment together with CMT	1999~	48,000 USD every year	
Scholarship	Chinese Government Scholarships	1950s~	254,000 USD every year recently	Support about 70 Nepalese medical students to study in China

Details in Table 2 also show the large parts of efforts from India’s DAH programs to facility and capacity building. In addition to that, India’s DAH programs in Nepal also contain public health interventions such as goiter control and the treatment of eye diseases.

**Table 2:** **Forms of India’s DAH programs in Nepal**

**Forms**	**Name of project**	**Period**	**Funding**	**Output**
Health facility building	Paropakar Maternal and Children Hospital	1959–1990s	NA	outpatient building, pathology laboratory, residents’ research center and ICU
	Bir Hospital	1984–1985	NA	5-floor outpatient building and nuclear medical center
	Emergency & trauma center	2003–2014	23 million USD	200-bedded and 8-floor building
	BPKIHS	1993–1999	21 million USD	50 seat medical college and 350-bedded afflicted hospital
	Teaching block in BPKIHS	2010–2013	1.2 million USD	4 large classrooms and 5 laboratories
	clinics	1973–1975	2.3 million USD	12 clinics
	Small development project	2003~	6 million USD	21 clinics and nursing campus in 17 zones
Dispatch faculty	BPKIHS	1994~	890,000 USD every year	211 health professionals in 20 years
Donation of equipment	Ambulance	1994~	NA	342 ambulances to 70 districts in Nepal during 1994–2012
Disease intervention	Goitre Control Programme	1973–1998; 2005–2007; 2011–2012	Around 14.4 million USD	Support the salt company for iodization, package, transportation, promotion, and building warehouse.
	Cataract and Trachoma surgeries programme	2001~	5 million USD	financial support for 300–400 surgeries conducted by Nepal Eye Foundation every year
Scholarship	Colombo Plan, Golden Jubilee, Compex Nepal & Ayush Scholarship	1950s; 2002-, 2005-	202,000 USD every year recently	Scholarships for 50 MBBS in the Golden Jubilee Scholarship, 20 B. Pharma in the Compex Nepal scholar, 6 Ayush Scholarships and several others in the Colombo Plan or ITEC Scholarship

Tables 1 and 2 indicate that both China and India provide a large amount of DAH to health facilities construction in Nepal. The health facilities were usually turn-key projects paid by Chinese or Indian government and constructed by Chinese, Indian or sometimes Nepalese companies. These facilities were handed over to the Nepal government, and thereafter operated by the Nepalese, although sometimes still with support from China or India, such as capacity building and equipment support [government officer 20130802]. However, India has a longer history of health facilities building than China, and the building forms are more diversified, including not only large hospitals but also clinics and nursing campuses provided by small development projects. These facilities “*benefit the rural population of Nepal and promote primary health care in the country*” [Project manager 20130731p]. As the aided facilities are public institutes, they also “*improve the accessibility and affordability of health services in Nepal*” [Project manager 20130801].

There is slight difference between China and India when they contribute to the capacity building of the Nepalese in relation to medical science. Both China and India have dispatched health professionals to Nepal to train Nepalese doctors. Indian faculties help to educate medical students to become general practitioners at BPKIHS, while Chinese doctors train general practitioners to become specialists at the Cancer Hospital through hands-on training. After more than ten-year support from China and India, “*some young doctors in Cancer Hospital at that time have been outstanding experts in Nepal now*” [Project manager 20130809] and BKKIHS has trained “*more than 2200 health worker for the eastern Nepal*” [Project manager20130806]. Although both of the focuses are important in Nepal, it is more necessary for Nepal to obtain more general practitioners in rural areas, given the urgent shortage of these reported in the mid-term assessment of NHSP II [22]. In addition, both China and India provide scholarships for Nepalese students to study medicine in China and India, In the 1950s and 1960s, a large amount of Nepalese doctors got training in India by support from the Colombo Plan [Development partner 20130813p1]. This trend continues now as Nepal and India share the same medical system. Although China offers less scholarship than India, 70% of China’s governmental scholarship for Nepalese is given to the medical students and the Nepalese are willing to learn medicine in China for the low tuition fees [ Project manager 20130812].

Alongside health system strengthening, India’s DAH to Nepal also includes disease-specific interventions such as the Goiter Control Program and the Cataract and Trachoma Surgeries Program. In a recent evaluation, the Goiter Control Program was found to have increased household iodized salt coverage from 95% to 99.8% and adequate iodized salt coverage from 57.7% to 77% during 2005–2007 [23]. The eye camp screened 425,000 potential patients and cured 870,000 cataract patients, as well as 3900 trachoma patients, between 2001 and 2012 [24]. In addition, Cataract and Trachoma Surgeries Program is India’s only program supporting grass roots NGOs in Nepal, while China has yet to touch this area.

### The aid effectiveness of DAH given by China and India

Initiated by the Paris Declaration, evaluating aid effectiveness includes five principles: ownership, alignment, harmonization, managing for results and mutual accountability. Here we will mostly concern about ownership, alignment and harmonization.

As development partners of Nepal, China’s and India’s DAH programs in the country are mostly “recipient driven”, something which reflects the needs of Nepal and respects its ownership. The Nepalese Ministry of Finance usually “*initiated a list of projects in need to Chinese and Indian government to require for help*”, [government officer 20130729a1] while China and India chose some of the projects to implement.

Both China and India contributed to Nepal’s Second Long Term Health Plan (1997–2017) [government officer 20130728p1]. They did so by providing “essential curative services for the appropriate treatment of common diseases and injuries”, and “the appropriate numbers, distribution and types of technically competent and socially responsible health personnel for quality healthcare throughout the country” [25], along with health facilities and capacity building, as well as medicine and equipment donations. Moreover, India’s support for the Nepal Eye Foundation also “develops appropriate roles for NGOs in providing health services” [25]. What China and India are doing in Nepal meets the “alignment” principle of aid effectiveness.

However, even if China’s and India’s development assistance for health in Nepal meets the principle of alignment, negotiations over such cooperation are still bilateral. Therefore, they may sometimes be influenced by political processes rather than the needs of the general population When China and India implement health programs in Nepal they usually “*do not coordinate with other external development partners in Nepal*” [Development partner 20130802a1]. This may affect the harmonization of DAH in Nepal and challenge the Nepalese government’s capacity to coordinate among its development partners. Both India and China need to participate in the broader partnership platform in order to adhere to the development cooperation partnership framework as agreed in the Busan Declaration [Development partner 20130813p1].

## Discussion

### Both China and India have a long history of providing DAH to Nepal and the amount of aid is increasing

China and India started providing DAH to Nepal in the 1950s. During the last sixty years of development assistance, both of these two giants have gradually made health a priority for development assistance. This is partly because of the rapid economic development of China and India during the last 20 years, which has resulted in growing amounts of total development assistance being made available. Viewed from the Nepalese perspective, Nepal has received development assistance and cultural influence from western countries for decades. These countries have influenced the government of Nepal to recognize the importance of livelihood development and to request China and India to help improve the health conditions of the Nepalese. In response to Nepal’s requests, China and India have reinvested and increased their DAH to Nepal.

**Figure 3:**
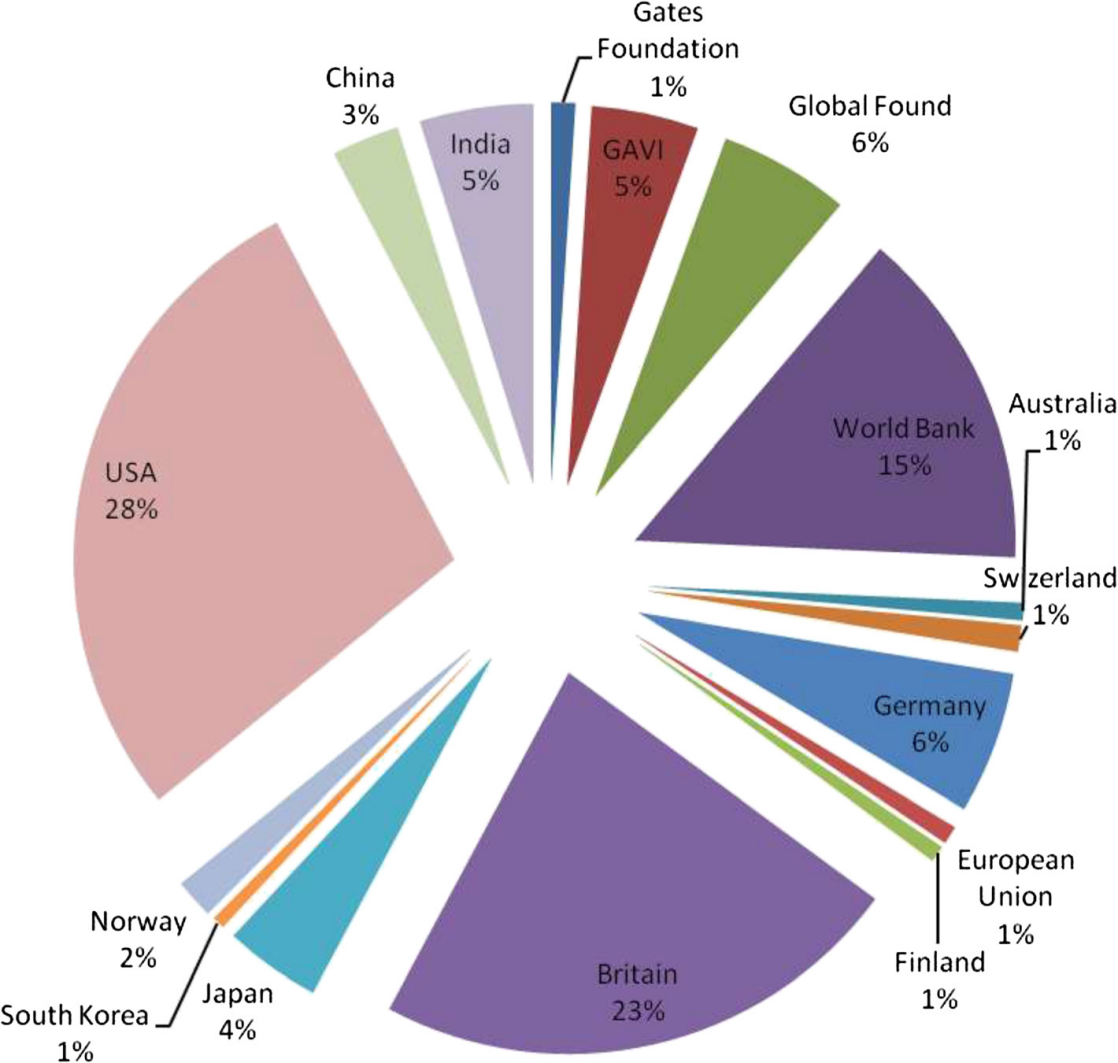
**Development assistance for Health from different donors to Nepal (2000–2009).** Source: Institute for Health Metrics and Evaluation (IHME). Development Assistance for Health Database 1990–2011. Seattle, United States: Institute for Health Metrics and Evaluation (IHME), 2014. Data for China and India come from calculating the sum of project funding in Tables 1 and 2.

Although DAH from China and India is increasing, the main health donors in Nepal are OECD-DAC members and multilateral organizations [26], while China and India contributed to around 3% and 5% of the total DAH to Nepal in 2000–2009. The USA and Britain were the largest bilateral donors of Nepal, while World Bank plays as the main multilateral health donor in Nepal (Figure 3). DAH funding from China and India to Nepal is at the same level of the traditional donors such as Germany, Japan and Norway. In addition, DAH from China and India to Nepal is an appropriate supplement to the OECD donors’ programs in Nepal (see section below) and has strategic importance. As “a yam between two boulders”, Nepal is a strategic area between China and India. India views Nepal as a strategic buffer against China, while China does not expect Nepal to be a pawn of the US and to allow India to encircle China [27]. Both China and India are prepared to use development assistance as a tool to safeguard national security, but less for the economic or trade interest. Nepal also makes full use of the competition between China and India to obtain more external development aid with the use of “balance diplomacy” [28].

### The main forms of DAH from China and India to Nepal are facilities and capacity building, which strengthen the health system of Nepal and supplement traditional donors’ contributions to the country

As initiators of the Five Principles of Peaceful Coexistence^b^, China and India maintain an equal development partnership with Nepal which respects the “ownership” of Nepal. Hence, they do not interfere with the inner affairs of health development planning in Nepal. Five Principles of Peaceful Coexistence initiated in the 1950s are fundamental principles for foreign policy and aid policy in both China and India from then on, indicating that south-south cooperation in development assistance has realized and promoted the effectiveness of aid earlier than the north donors. All programs relating to DAH from China and India have been agreed based on a recipient-driven mechanism in which Government of Nepal makes aid requests to the embassies of China and India in Kathmandu. This mechanism ensures that all such programs meet the urgent needs of Nepal. This non-interference in internal affairs, unconditional aid and equal development partnership reflect the features of south-south cooperation.

While the majority of the western countries emphasize public health intervention or pooled funding support in Nepal, Nepal resorts to China’s and India’s turn-key projects for facilities building to improve the health sector’s infrastructure. With the priorities of different donors being effectively designed by the Nepalese government, the two neighbors’ DAH programs are an appropriate supplement to the OECD donors’ programs in Nepal.

### India’s DAH not only supports the health system but also some key disease interventions

Even though both China and India are southern donors, differences still exist between them. The geography of North India is similar to Nepal, which makes India’s experience of goiter control easier to apply in Nepal. India and Nepal also have the similar language, religion, culture, and health system to make it easier for India’s experience to transfer to Nepal. However, the reasons for India’s unique public health interventions in Nepal are not really that simple. Diplomatic interests may contribute to different forms of DAH in different countries. As India’s foreign policy emphasizes South Asia and neighboring countries, India’s DAH programs in Nepal are different from those in countries outside South Asia. India would not be prepared to build facilities and implement public health interventions in other countries outside South Asia. China, in contrast, adopts similar forms of DAH for all developing countries as development partners.

Whatever their form, however, public health interventions are as important as medical services when fighting diseases. China has accumulated experience in disease control and prevention which can be transferred to Nepal, as is the case for India. The effects of public health intervention with China’s support may be better than the effects of providing medical service at present.. This is especially true at present, when Nepal is in need of relevant experience in non-communicable disease (NCD) control as the risks and burden of NCDs have recently been increasing.

### India is trying to strengthen cooperation with NGOs and local government in Nepal while maintaining its traditional relationship with the central government of Nepal

A typical feature of Nepalese culture is inclusiveness. The coexistence of different cultures in Nepal encourages the Nepalese to accept different external assistance and experiences from western countries [29]. The influence of western aid and culture has promoted the recognition of civil rights among the Nepalese and the development of thousands of grass roots NGOs in Nepal. They work together with the local government and contribute to local health development. To adapt to this change, the Indian government has started to cooperate with Nepalese NGOs and local government. Although the local NGO in Nepal highly valued India’s support and propagate for public health intervention from China and India, the Nepal government seems to be unknown about India’s small projects in local Nepal and wish large projects from the two neighbors. Besides, the government of China may not directly donate to grass roots NGOs in Nepal, as grass roots NGOs in China start to develop recently. It really needs time for the Chinese government to understand the importance of grass roots NGOs. But the government could encourage the government owned NGOs and academic institutes in China to provide technical assistance for local health development in Nepal, such as academic exchanges and dispatching volunteers. Such cooperation with local people will make China’s aid more appropriate for the Nepalese.

## Conclusion

As the neighboring countries of Nepal and emerging DAH donors, China and India work as equal development partners of Nepal and respect its ownership while providing DAH. The Five Principles of Peaceful Coexistence initiated by China and India have determined a recipient-driven mechanism, the priorities set and increases in DAH. These principles keep the two countries’ DAH in line with Nepal’s health development plan and ensure they obey the ownership and alignment principles of aid effectiveness.

However, the more diversified forms reflected in public health interventions and cooperation with NGOs and local government in India’s programs seem to be closer to the needs of the rural population. Since there are many similarities between Northeastern India and Nepal in terms of culture and geography, India’s DAH has incorporated its experience in public health intervention and working with NGOs as part of its government’s diplomatic priorities. China could learn from India by disseminating its experience in disease control and cooperating with NGOs through DAH.

### Consent

Written informed consent was obtained from the participants for the publication of this report and any accompanying images/data.

## Endnotes

^a^The snowball method involves interviewing one important person in this field and gaining contact with others through introductions made by this person. This method was used on an individual basis to obtain the number of interviewees needed.

^b^Five Principles of Peaceful Coexistence: mutual respect for sovereignty and territorial integrity, mutual non-aggression, non-interference in each other’s internal affairs, equality and mutual benefit, and peaceful coexistence.
